# Tumor suppressor death-associated protein kinase 1 inhibits necroptosis by p38 MAPK activation

**DOI:** 10.1038/s41419-020-2534-9

**Published:** 2020-05-04

**Authors:** Yung-Hsuan Wu, Ting-Fang Chou, Leslie Young, Fu-Yi Hsieh, Hsuan-Yin Pan, Shu-Ting Mo, Shani Bialik Brown, Ruey-Hwa Chen, Adi Kimchi, Ming-Zong Lai

**Affiliations:** 1 Institute of Molecular Biology, Academia Sinica, Taipei, 11529 Taiwan; 2 Department of Molecular Genetics, Weizmann Institute of Science, Rehovot, 76100 Israel; 3 Institute of Biological Chemistry, Academia Sinica, Taipei, 11529 Taiwan

**Keywords:** Immune cell death, Inflammation, Signal transduction, Inflammation

## Abstract

Death-associated protein kinase 1 (DAPK1, DAPk, DAPK) is known for its involvement in apoptosis and autophagy-associated cell death. Here, we identified an unexpected function of DAPK1 in suppressing necroptosis. DAPK1-deficiency renders macrophages and dendritic cells susceptible to necroptotic death. We also observed an inhibitory role for DAPK1 in necroptosis in HT-29 cells, since knockdown or knockout of DAPK1 in such cells increased their sensitivity to necroptosis. Increased necroptosis was associated with enhanced formation of the RIPK1–RIPK3–MLKL complex in these DAPK1-deficient cells. We further found that DAPK1-deficiency led to decreased MAPK activated kinase 2 (MK2) activation and reduced RIPK1 S321 phosphorylation, with this latter representing a critical step controlling necrosome formation. Most TNF signaling pathways, including ERK, JNK, and AKT, were not regulated by DAPK. In contrast, DAPK bound p38 MAPK and selectively promoted p38 MAPK activation, resulting in enhanced MK2 phosphorylation. Our results reveal a novel role for DAPK1 in inhibiting necroptosis and illustrate an unexpected selectivity for DAPK1 in promoting p38 MAPK-MK2 activation. Importantly, our study suggests that modulation of necroptosis and p38/MK2-mediated inflammation may be achieved by targeting DAPK1.

## Introduction

Necroptosis is typically induced in cells via activation of the receptor-interacting protein kinase 1 (RIPK1)-RIPK3 cascade and concomitant inactivation of the FADD–caspase-8–c-FLIP complex^1–6^. Necroptosis is triggered by a wide variety of stimuli including tumor necrosis factor (TNF), Toll-like receptor agonists, second mitochondria-derived activator of caspases (SMAC) mimetics, interferons (IFNs), microbial infection, or DNA-damaging chemicals^7^. Binding of TNF to TNF receptor 1 (TNFR1) leads to assembly of complex I^8^ at the plasma membranes by recruitment of TNF receptor-associated protein with death domain (TRADD) and RIPK1, followed by TNFR-associated factor 2 (TRAF2), TRAF5, cellular inhibitors of apoptosis 1 (cIAP1) and cIAP2, and the linear ubiquitin chain assembly complex (LUBAC). The E3 ligases cIAP1/2 add K63 polyubiquitin chains to RIPK1 to recruit the transforming growth factor beta-activated kinase 1 (TAK1) and IκB kinase (IKK) complexes and induce NF-κB activation, whereas LUBAC introduces M1-linked polyubiquitin chains to RIPK1 and other complex I proteins for signaling. Loss of RIPK1 ubiquitination by depletion of cIAP1/2 or LUBAC or by deubiquitination through cylindromatosis protein (CYLD), together with association of FADD and caspase-8, result in complex II formation. Inactivation or depletion of caspase-8 converts pro-apoptotic complex II into a pro-necroptotic complex, with RIPK1 binding and activating RIPK3^9–11^, resulting in RIPK3 auto-phosphorylation and activation of mixed lineage kinase domain-like (MLKL)^12,13^. Phosphorylated MLKL is translocated into the plasma membranes and wherein forms pores that lead to membrane leakage and necroptotic cell death^14–16^. Necroptosis associated with depletion of FADD, caspase-8, or c-FLIP can be prevented by concomitant deletion of RIPK1 or RIPK3^17–19^. In contrast, SMAC mimetics (used to promote cIAP1/2 degradation) and zVAD (a pan-caspase-inhibitor used to inactivate caspase-8) can induce necroptosis.

Recent studies have further revealed several cell death suppressing checkpoints centered on RIPK1. Complex I-associated RIPK1 is subjected to phosphorylation by IKK that inhibits the transition into complex II^20^. In a different inhibitory process, TNF-induced activation of p38 MAPK leads to phosphorylation of MAPKAPK2 (MK2), with this latter directly phosphorylating RIPK1 at the S321 and S326 positions (in mouse; S320 and S325 in human) to prevent binding of RIPK1 with FADD/caspase-8 to form complex II^21–23^. Alternatively, TBK1-mediated phosphorylation of RIPK1 inhibits RIPK1 activation and the conversion into complex II^24,25^.

Necroptosis participates in numerous pathological events^3–6,26^. It often leads to inflammation and is one of the mechanisms involved in counteracting specific viral infections, while expression of viral molecules can result in evasion of the necroptotic process. Necroptosis also contributes to ischemia-reperfusion injury, transplantation rejection, and cancer inhibition or progressing^3–6,26^.

Death-associated protein kinase 1 (DAPK, DAPk, DAPK1) is a multi-domain serine/threonine kinase regulated by calcium^27–29^. It was first identified for its role in mediating IFN-γ-induced cell death^30^, but subsequent evidence demonstrated involvement in apoptotic cell death induced by Fas^31^, TGF-β^32^, ceramide^33^, matrix detachment^34^, unliganded Netrin-1 receptor uncoordinated protein 5 homolog 2 (UNC5H2)^35^, or ER stress^36^. Although how DAPK1 regulates apoptosis remains incompletely understood^37^, it is known that it interacts with p53 and promotes p53-dependent cell death^38,39^. In addition, DAPK1 interacts with Fas-associated protein with death domain (FADD)^38^. DAPK1 is a tumor suppressor and is specifically downregulated in many types of cancer^40,41^. Apart from its role in apoptosis, DAPK1 participates in a wide variety of cellular events including autophagy, membrane blebbing, and stress fiber formation that all contribute to its tumor-suppressing functions. In T lymphocytes, DAPK1 inhibits T cell activation by suppressing T cell receptor-induced NF-κB activation^42^.

In the present study, we found that DAPK1 negatively regulates necroptosis, unlike its active involvement in other forms of cell death. DAPK1-deficiency enhances the sensitivity of myeloid and HT-29 cells to necroptotic induction. Knockdown or knockout of DAPK1 in HT-29 cells increases their sensitivity to necroptosis. The increased necroptosis in DAPK1-deficient cells was associated with enhanced formation of the RIPK1–RIPK3–MLKL complex. We further found that DAPK1 selectively increased TNF-induced p38 MAPK and MK2 activation, leading to phosphorylation of RIPK1 at position S321 and inhibition of necrosome formation. Our results reveal a novel role for DAPK1 in inhibiting necroptosis and illustrate the diverse death-associated physiological functions regulated by DAPK1.

## Results

### DAPK1-deficiency sensitizes myeloid cells to necroptosis

**Fig. 1:**
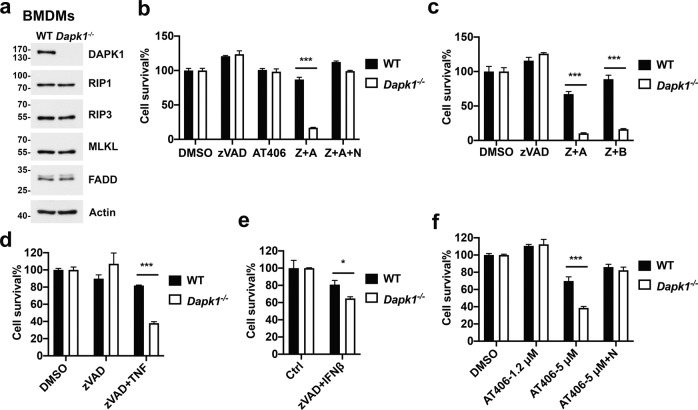
DAPK1-deficient BMDMs are more sensitive to necroptotic induction. **a** DAPK1 deficiency does not affect expressions of FADD, RIPK1, RIPK3, or MLKL in BMDMs. **b**, **c** *Dapk1*^−*/−*^ BMDMs exhibit increased cell death induction relative to WT upon zVAD+AT-406 treatment. WT and *Dapk1*^*−/−*^ BMDMs were stimulated with DMSO, AT-406 (0.6 μM, A), zVAD (20 μM, Z), Nec-1 (40 μM, N), or BV6 (0.5 μM, B), as indicated, for 18–20 h, before determining cell death according to release of ATP. **d**, **e** zVAD+TNF or zVAD+IFN-β treatments trigger increased necroptosis in *Dapk1*^*−/−*^ BMDMs. WT and *Dapk1*^*−/−*^ BMDMs were treated with zVAD + TNF (5 ng/ml) (**d**) or zVAD + IFN-β (5 ng/ml) (**e**) and then cell viability was determined. **f** High dose of AT-406 induces necroptosis. WT and *Dapk1*^*−/−*^ BMDMs were treated with AT-406 at the indicated dose, without or with Nec-1, and cell viability quantitated. Values are mean ± SD of triplicates in a single experiment. *P < 0.05, **P < 0.01, ***P < 0.001 for unpaired *t*-test. Data have been repeated in two (**e**, **f)** or three (**a**–**d**) independent experiments.

We used bone marrow-derived macrophages (BMDMs) from wild-type control (WT) and *Dapk*^*−/−*^ mice to examine the possible role of DAPK1 in necroptosis. DAPK1 knockout did not affect the development of myeloid cells in bone marrow or spleen (Supplementary Fig. 1), nor did DAPK1 deficiency affect the protein expression of RIPK1, RIPK3, MLKL, or FADD in BMDMs (Fig. 1a). Treatment of BMDMs with the SMAC mimetic AT-406 or the pan-caspase inhibitor zVAD alone did not affect macrophage viability, as measured by release of ATP (Fig. 1b, c). However, a combination of zVAD and AT-406 induced cell death in BMDMs, which was suppressed by the inclusion of RIPK1 inhibitor necrostatin-1 (Nec-1), confirming its necroptotic nature (Fig. 1b). Unexpectedly, DAPK1-deficient BMDMs were much more sensitive to cell death induced by zVAD plus AT-406 than WT BMDMs (Fig. 1b). We observed a similar necroptotic outcome in *Dapk1*^*−/−*^ BMDMs when we used zVAD together with another SMAC mimetic, BV6 (Fig. 1c). In addition, DAPK1-deficient macrophages exhibited higher sensitivity to necroptotic death triggered by zVAD plus TNF or zVAD plus IFN-β^7^ (Fig. 1d, e). We also tested the sensitivity of *Dapk1*^*−/−*^ BMDMs to SMAC mimetic alone in the absence of zVAD. At higher dose (5 μM), AT-406 triggered necroptosis which was significantly enhanced by DAPK1 deficiency (Fig. 1f). We also measured cell viability according to incorporation of MTT (3-(4,5-dimethylthiazol-2-yl)-2,5-diphenyl tetrazolium bromide) (Supplementary Fig. 2), which showed that treatments with zVAD+AT-406 lowered the viability of *Dapk1*^*−/−*^ BMDMs compared to WT BMDMs, but addition of Nec-1 effectively restored cell viability. We observed similar findings in bone marrow-derived dendritic cells (Supplementary Fig. 3).

We then knocked down DAPK1 in the mouse macrophage cell line J774A.1 (Supplementary Fig. 4a). By itself, zVAD (20 μM) triggered weak necroptosis in WT J774A.1 cells (Supplementary Fig. 4b), but this effect was markedly enhanced upon DAPK1 knockdown, and Nec-1 addition completely attenuated the impact of DAPK1 knockdown (Supplementary Fig. 4b). We could induce significant cell death in DAPK1-knockdown J774A.1 cells via a combination of a low concentration of zVAD (5 μM) plus AT-406 (Supplementary Fig. 4c). Furthermore, re-introduction of DAPK1 to DAPK1-deficient J774A.1 cells rescued them from the necroptosis triggered by zVAD+AT-406 (Supplementary Fig. 4c), confirming the specific involvement of DAPK1 in this process^43^.

In contrast, we found that *Dapk1*^*−/−*^ BMDMs were more resistant to thapsigargin-triggered apoptosis than WT BMDMs (Supplementary Fig. 5a), consistent with the pro-apoptotic role of DAPK1 in ER stress-induced cell death^36^. In Jurkat cells, a cell line sensitive to Fas-initiated apoptosis, DAPK1 knockdown did not affect surface Fas expression but it did reduce Fas ligand (FasL)-triggered cell death (Supplementary Fig. 5b, c). BMDMs are moderately sensitive to FasL-induced apoptosis, and we found that DAPK1-deficiency reduced the extent of cell death mediated by FasL in such cells (Supplementary Fig. 5d). Therefore, consistent with the known involvement of DAPK1 in apoptosis, DAPK1-deficiency attenuates ER stress- and FasL-induced cell death. The enhanced susceptibility of DAPK1-deficient myeloid cells to necroptosis reveals a selective inhibitory role for DAPK1 in necroptosis.

### Necroptosis is increased upon downregulation of DAPK1 in HT-29 cells

**Fig. 2:**
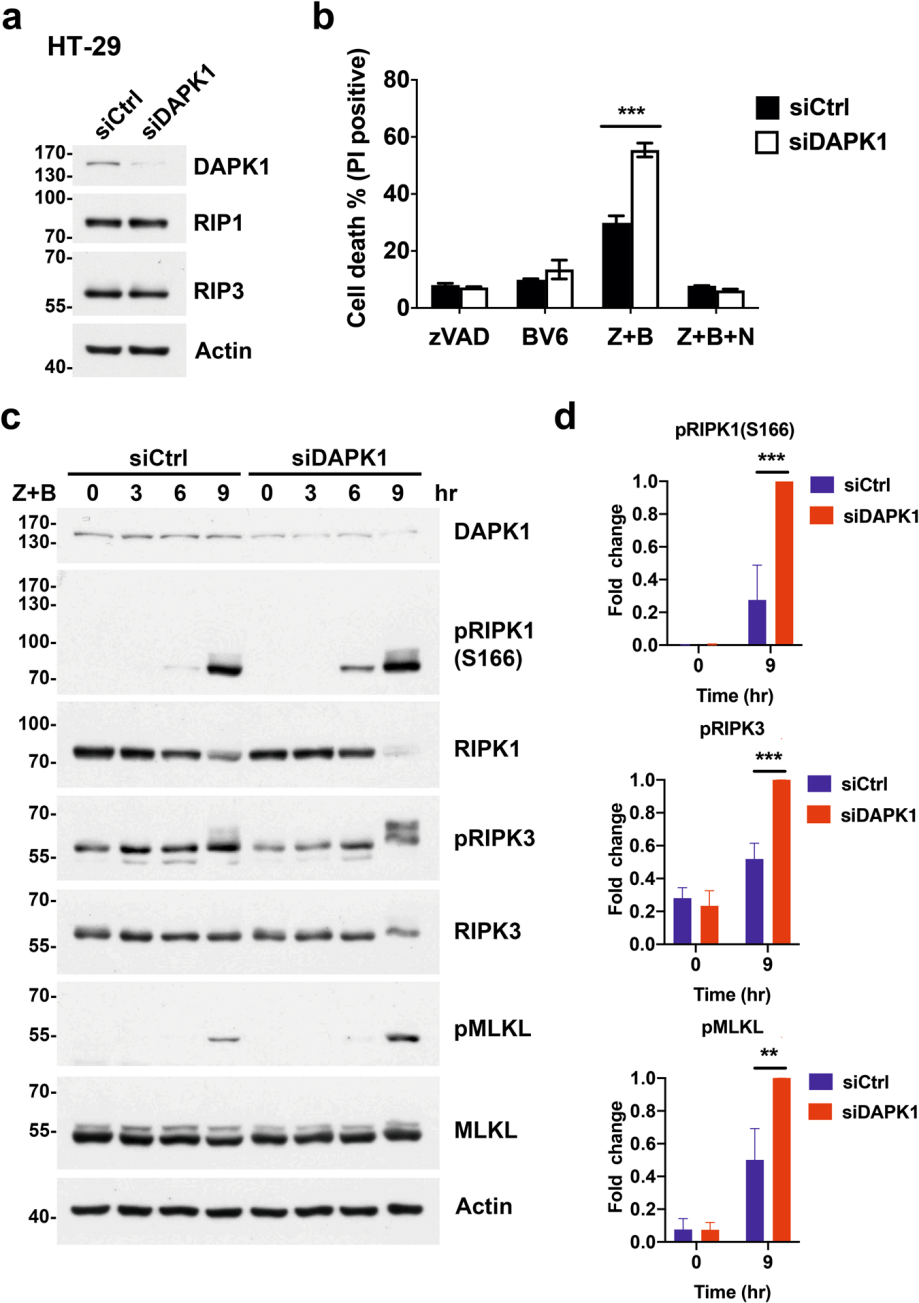
Knockdown of DAPK1 confers increased sensitivity to necroptosis on HT-29 cells. **a** Knockdown of DAPK1 in HT-29 cells. HT-29 cells were transduced with pLL3.7-shCtrl or pLL3.7-shDAPK1, sorted, and then expressions of DAPK1, RIPK1 and RIPK3 were determined. **b** Increased zVAD+BV6-induced necroptosis in DAPK1-deficient HT-29 cells. Control and DAPK1-knockdown HT-29 cells were treated with zVAD (20 μM), BV6 (1 μM), Nec-1 (40 μM) as indicated for 24 h, cell death was quantitated by PI staining. Values are mean ± SD of triplicates in a single experiment. ***P < 0.001 for unpaired *t*-test. Results have been confirmed in three independent experiments. **c** Enhanced zVAD+BV6-induced phosphorylation of RIPK1, RIPK3 and MLKL in DAPK1-deficient HT-29 cells. Control and DAPK1-knockdown HT-29 cells were treated with zVAD+BV6 (0.5 μM, Z + B) and contents of RIPK1, pS166-RIPK1, RIPK3, pS227-RIPK3, MLKL, and pMLKL in cell lysates were determined at the indicated time points. Right panel, quantitation of pRIPK1(S166), pRIPK3 and pMLKL from three independent experiments using normalized intensity of pRIPK1(S166), pRIPK3 and pMLKL in DAPK1-knockdown HT29 cells at 9 h as 1. **P < 0.01, ***P < 0.001 for two-way ANOVA followed by a Tukey’s multiple comparison test.

The enhanced sensitivity to necroptosis was not restricted to *Dapk1*^*−/−*^ myeloid cells. A similar effect was found in the human colon adenocarcinoma cell line HT-29. HT-29 cells were previously shown to be susceptible to necroptotic induction by treatment with zVAD plus SMAC mimetics^9^. We knocked down DAPK1 by shRNA in HT-29 cells, which did not affect expression of RIPK1 or RIPK3 (Fig. 2a). Treatment of WT HT-29 cells with zVAD or BV6 alone did not trigger cell death, as measured by propidium iodide (PI) staining (Fig. 2b). However, a combination of zVAD plus BV6 did induce cell death in WT HT-29 cells, and this outcome was significantly enhanced upon DAPK1 deficiency (Fig. 2b). The necroptotic nature of the cell death outcome was confirmed by addition of Nec-1, which suppressed the effect in both WT and DAPK1-knockdown HT-29 cells (Fig. 2b).

We also generated DAPK1-knockout HT-29 cells by CRISPR-Cas9 editing (Supplementary Fig. 6a) and found that expression of necroptosis-associated molecules (RIPK1, RIPK3, MLKL, and FADD) was comparable between WT and DAPK1-knockout HT-29 cells (Supplementary Fig. 6b). The necroptosis induced by zVAD+BV6 was also significantly enhanced in these DAPK1-knockout HT-29 cells and, importantly, it was inhibited by Nec-1 addition (Supplementary Fig. 6c). Therefore, DAPK1-deficiency enhances necroptotic induction in macrophages, dendritic cells and HT-29 cells, supporting the inhibitory role of DAPK1 in necroptosis.

### Overexpression of DAPK1 inhibits necroptosis

We next generated HT-29 cells that overexpress DAPK1 (Supplementary Fig. 7a). The necroptosis induced by zVAD plus TNF treatment was attenuated in DAPK1-overexpressing HT-29 cells relative to vector control (Supplementary Fig. 7b). We were also able to recapitulate the anti-necroptotic effect of DAPK1 by re-introducing it into DAPK1-null HT-29 cells (Supplementary Fig. 7c, d). Together, these results further validate that DAPK1 suppresses necroptosis.

### Increased phosphorylation of RIPK1(S166), RIPK3, and MLKL in DAPK1-deficient cells

**Fig. 3:**
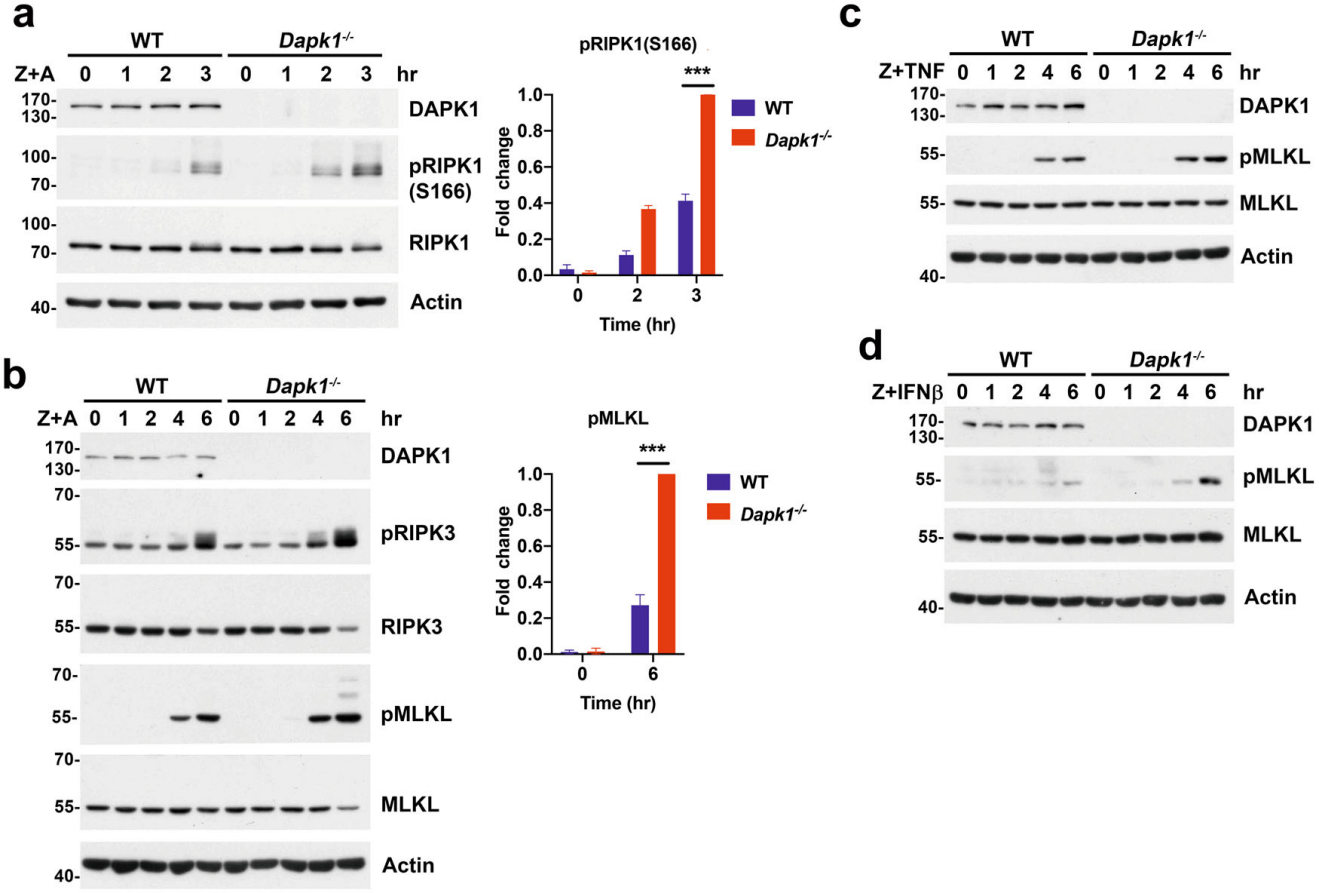
Enhanced activation of RIPK1, RIPK3 and MLKL in DAPK1-deficient cells after necroptotic induction. **a**, **b** Increased zVAD+AT-406-induced phosphorylation of RIPK1, RIPK3 and MLKL in *Dapk1*^*−/−*^ BMDMs. WT and *Dapk1*^*−/−*^ BMDMs were treated with zVAD (20 μM) plus AT-406 (0.6 μM, Z + A), and cell lysates were prepared at the indicated time points. The contents of RIPK1, pS166-RIPK1 (**a**), or RIPK3, pRIPK3, MLKL, and pMLKL (**b**) were determined by Western blot. Right panel, quantitation of pRIPK1(S166) (**a**) and pMLKL (**b**) from three independent experiments using normalized intensity of pRIPK1(S166) (3 h) and pMLKL (6 h) in *Dapk1*^*−/−*^ BMDMs as 1. ***P < 0.001 for two-way ANOVA followed by a Tukey’s multiple comparison test. **c**, **d** Increased activation of MLKL in *Dapk1*^*−/−*^ BMDMs stimulated by treatment with zVAD plus TNF or IFN-β. WT and *Dapk1*^*−/−*^ BMDMs were treated with zVAD in combination with TNF (**c**), or IFN-β (**d**) and levels of MLKL and pMLKL in cell lysates were determined at the indicated time points. Data are representative of three independent experiments.

Apart from assessing cell death and viability, we also examined certain biochemical events associated with necroptosis in our *Dapk1*^*−*/−^ cells. During TNF-initiated necroptotic signaling, RIPK1 is first phosphorylated at S166, before the RIPK1-mediated activation of RIPK3 that induces MLKL activation and cell death. Treatment of BMDMs with zVAD+AT-406 induced activation of RIPK1, RIPK3, and MLKL (Fig. 3a, b). We also observed increased phosphorylation of RIPK1(S166), RIPK3 and MLKL in *Dapk1*^*−*/−^ BMDMs relative to WT BMDMs upon treatment with zVAD+AT-406 (Fig. 3a, b). Moreover, we detected enhanced MLKL phosphorylation in *Dapk1*^*−*/−^ BMDMs upon treatment with zVAD plus either TNF or IFN-β (Fig. 3c, d).

Similarly, RIPK1(S166), RIPK3, and MLKL all displayed increased phosphorylation in both DAPK1-knockdown and DAPK1-knockout HT-29 cells (relative to WT HT-29 cells) after zVAD+BV6 combinatory treatment (Fig. 2c, d, Supplementary Fig. 6d). Therefore, DAPK1-deficient cells exhibit enhanced activation of the RIPK1-RIPK3-MLKL axis, which is consistent with their increased necroptotic activity.

### Increased association of RIPK1 with FADD and caspase-8 in DAPK1-deficient cells

**Fig. 4:**
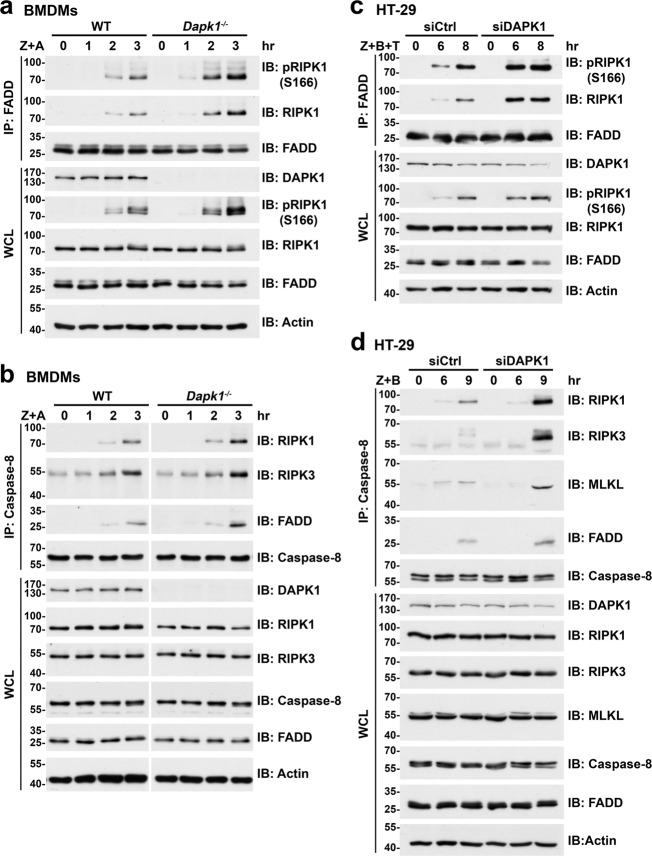
Enhanced associations of RIPK1 and RIPK3 with FADD or caspase-8 in DAPK1-deficient cells. **a**, **b** Enhanced zVAD+AT-406-induced binding of RIPK1 and RIPK3 to FADD/caspase-8 in *Dapk1*^*−/−*^ BMDMs. WT and *Dapk1*^*−/−*^ BMDMs were treated with zVAD+AT-406 (Z + A), and whole-cell lysates (WCL) were prepared at the indicated time points. WCL were immunoprecipitated with anti-FADD and contents of pRIPK1, RIPK1 and FADD in the precipitates and WCL were determined (**a**), or they were immunoprecipitated with anti-caspase-8 and the amounts of RIPK1, RIPK3, FADD and caspase-8 in the precipitates and WCL were determined (**b**). **c**, **d** Increased binding of RIPK1 and RIPK3 to FADD/caspase-8 in DAPK1-deficient HT-29 cells upon necroptotic induction. Control and DAPK1-knockdown HT-29 cells were treated with zVAD+BV6 + TNF (Z + B + T) (**c**) or zVAD+BV6 (Z + B) (**d**) before collecting WCL at the indicated time points. WCL were immunoprecipitated with anti-FADD and the contents of pRIPK1, RIPK1 and FADD in the precipitates and WCL were determined **(c**), or we immunoprecipitated them with anti-caspse-8 and the amounts of RIPK1, RIPK3, FADD and caspase-8 were determined in the precipitates and WCL (**d**). Data are representative of three independent experiments.

TNF-induced necroptosome formation begins upon binding of RIPK1 to FADD and caspase-8. We observed that immunoprecipitation of FADD brought down RIPK1 and RIPK1 phosphorylated at position S166 [pRIPK1(S166)] after BMDMs had been treated with zVAD+AT-406 (Fig. 4). However, DAPK1-deficiency increased the association of both RIPK1 and pRIPK1(S166) with FADD upon zVAD+AT-406 stimulation (Fig. 4a). Similarly, immunoprecipitation of caspase-8 pulled down RIPK1, RIPK3 and FADD in macrophages treated with zVAD+AT-406, and levels of caspase-8-bound RIPK1 and RIPK3 were higher in *Dapk1*^*−*/−^ BMDMs than WT counterparts after necroptosis induction (Fig. 4b).

We observed a comparable scenario for DAPK1-deficient HT-29 cells. Treatment of HT-29 cells with zVAD plus both BV6 and TNF led to stronger association of FADD with RIPK1 and pRIPK1(S166) (Fig. 4c). DAPK1-knockdown increased the binding of RIPK1 and pRIPK1(S166) to FADD. Immunoprecipitation of caspase-8 also brought down more RIPK1, RIPK3, MLKL, and FADD from DAPK1-deficient HT-29 cells than was the case for control HT-29 cells (Fig. 4d). Together, these results illustrate that DAPK1-deficiency enhances formation of the FADD–caspase-8–RIPK1–RIPK3–MLKL complex.

### Sensitization to TNF-induced septic shock in DAPK1-knockout mice

**Fig. 5:**
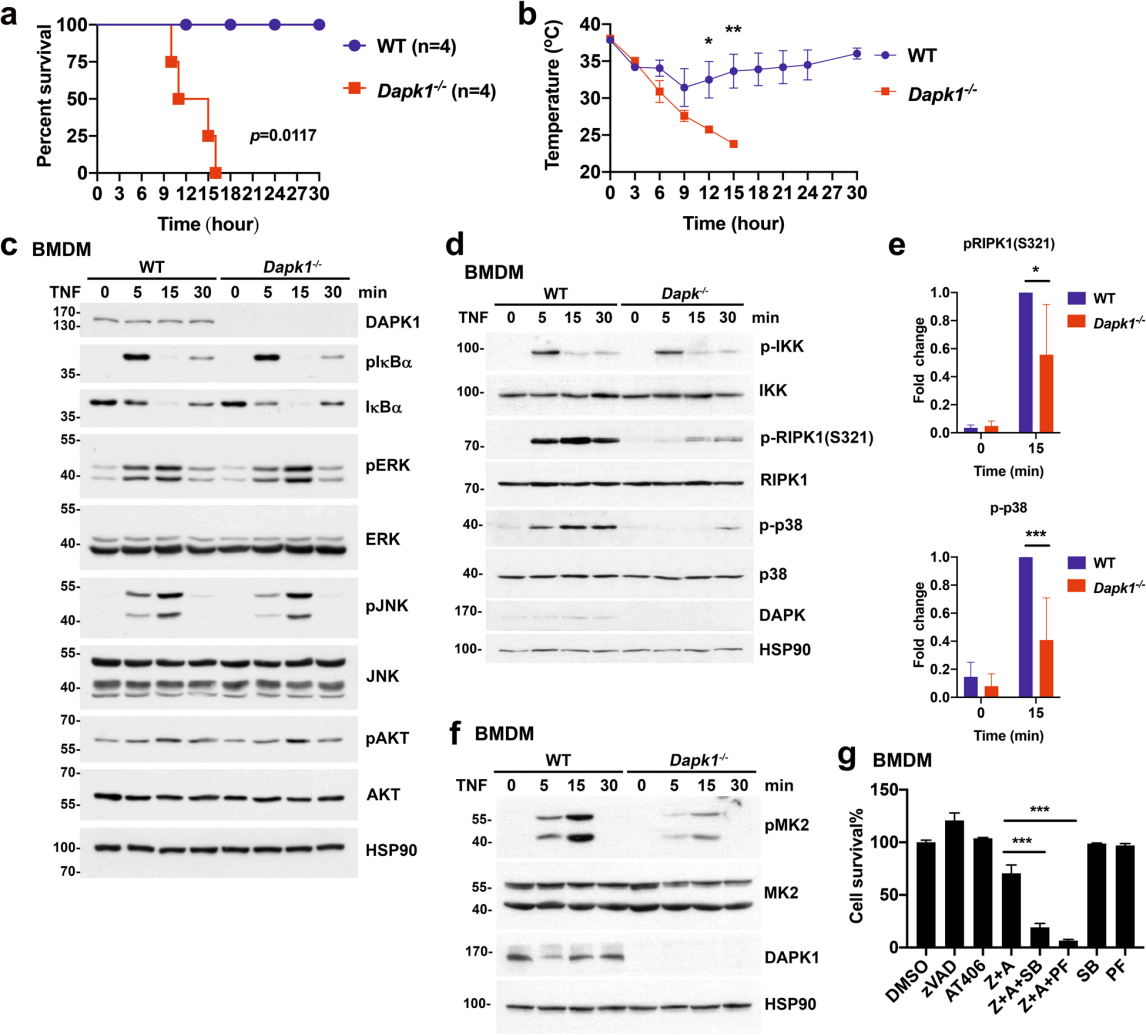
Sensitivity to TNF-induced septic shock in *Dapk1*^*−/−*^ mice and DAPK1-deficiency selectively inhibits phosphorylation of RIPK1(S321), MK2, and p38 MAPK. **a**, **b** Control and *Dapk1*^*−/−*^ mice (*n* = 4 in each group) were injected with mouse TNF (1.0 μg/g body weight), and survival (**a**) and rectal body temperature (**b**) were determined at the indicated time points. *P* values (**a**) for Long-rank (Mantel-–Cox) test. Mean ± SD (**b**) are shown. *P < 0.05, **P < 0.01 (**b**) for two-way ANOVA followed by a Tukey’s multiple comparison test. (**c**) Normal TNF-triggered activation or NF-κB, ERK, JNK, and AKT in *Dapk1*^*−/−*^ BMDMs. WT and *Dapk1*^*−/−*^ BMDMs were treated with TNF (20 ng/ml), and cell lysates were prepared and levels of pIκBα, IκBα, pERK, ERK, pJNK, JNK, pAKT, and AKT were determined for the indicated time points. (**d**, **f**) Diminished TNF-induced phosphorylation of RIPK1(S321), MK2 and p38 MAPK in *Dapk1*^*−/−*^ BMDMs. WT and *Dapk1*^*−/−*^ BMDMs were treated with TNF (20 ng/ml), and levels of pIKK, IKK, pRIPK1(S321), RIPK1, p-p38, p38 (**d**), pMK2 and MK2 (**f**) were determined in cell lysates for the indicated time points. Data are representative of three independent experiments. **(e**) The extents of RIPK1(S321) and p38 MAPK activation from three independent experiments in (**d**) were quantitated using normalized intensity of pRIPK1(S321) and p-p38 in WT BMDMs at 15 min as 1. Mean ± SD are shown. *P < 0.05, ***P < 0.001 for two-way ANOVA followed by a Sidak’s multiple comparison test. (**g**) Inhibition of p38 MAPK or MK2 confers susceptibility to necroptosis in WT macrophages. WT BMDMs were treated with zVAD, AT-406, SB203589 (1 μM) and PF3644022 (2 μM), as indicated, and the extent of necroptosis determined. Values are mean ± SD of triplicates in a single experiment. **p < 0.01, ***p < 0.001 for unpaired *t*-test. Results have been repeated in two independent experiments.

We also examined whether DAPK1-deficiency increases necroptosis in vivo. In RIPK1- and RIPK3-dependent processes, necroptosis mediates TNF-induced systemic inflammatory response syndrome. The administration of TNF-induced hypothermia but did not trigger death in WT mice (Fig. 5a, b). DAPK1-deficient mice were highly sensitive to severe hypothermia and lethality triggered by TNF (Fig. 5a, b). Therefore, DAPK1-deficiency conferred sensitivity to necroptosis in cultured cells and in vivo.

### DAPK1-deficiency does not affect major TNF-induced survival signaling

We next examined how DAPK1 inhibits TNF-mediated necroptosis by determining TNF-induced survival signaling in WT and *Dapk1*^*−*/−^ BMDMs. TNF-triggered IκBα phosphorylation and IκBα degradation was comparable between WT and *Dapk1*^*−*/−^ BMDMs (Fig. 5c). Similarly, activation of extracellular signal-regulated kinase (ERK) and JNK by TNF was practically indistinguishable between WT and *Dapk1*^*−*/−^ BMDMs. TNF also triggered similarly moderate AKT activation in both WT and *Dapk1*^*−*/−^ BMDMs (Fig. 5c). We also assessed if DAPK1 affected degradation of cIAP1 by the SMAC mimetics AT-406 and BV6. AT-406-mediated cIAP1 downregulation was comparable between WT and *Dapk1*^*−*/−^ BMDMs (Supplementary Fig. 8a), and DAPK1-knockdown did not affect cIAP1-degradation triggered by BV6 in HT-29 cells (Supplementary Fig. 8b). These results suggest that DAPK1 deficiency does not affect most TNF-induced survival signals nor alter cIAP-1 degradation.

### Enhanced necroptosis in DAPK1-deficient cells is associated with decreased MK2-directed S321 phosphorylation of RIPK1

Despite that the majority of TNF-induced survival signaling were not affected by DAPK1-deficiency (Fig. 5c), recent studies have revealed that kinases downstream to TNFR can regulate RIPK1-containing death complex assembly in unconventional ways. For instance, IKKα/IKKβ inhibits RIPK1-mediated death independently of NF-κB activation via direct phosphorylation of RIPK1^20^. Moreover, p38/MK2-mediated phosphorylation of RIPK1 at positions S321 and S326 inhibits binding of RIPK1 to FADD/caspase-8^21–23^. Accordingly, we examined activation of IKKα/IKKβ, p38 MAPK and MK2, as well as phosphorylation of RIPK1 at S321, in DAPK1-deficient macrophages following TNF stimulation. We found that TNF-triggered activation of IKKα/IKKβ was comparable between WT and *Dapk1*^*−*/−^ BMDMs (Fig. 5d), but we did find reduced activation of p38 MAPK and decreased S321 phosphorylation of RIPK1 in *Dapk1*^−/−^ BMDMs relative to WT (Fig. 5d, e). The reduced phosphorylation of p38 MAPK in *Dapk1*^*−*/−^ BMDMs was also visualized in image analysis (Supplementary Fig. 9). Consistent with attenuated phosphorylation of p38 MAPK and RIPK1(S321) in DAPK-deficient cells, MK2 activation was compromised in DAPK-deficient macrophages (Fig. 5f). These outcomes likely reflect the enhanced formation of FADD–caspase-8–RIPK1–RIPK3 complex in DAPK1-deficient cells (Fig. 4). In WT BMDMs, treatment with p38 MAPK inhibitor SB203580 or MK2 inhibitor PF3644022 sensitized BMDMs to necroptosis induced by zVAD+AT406 (Fig. 5g), to the levels observed in *Dapk1*^*−*/−^ BMDMs (Fig. 1). Therefore, the increased necroptosis observed in DAPK1-deficient cells could be partly attributable to decreased activation of p38 MAPK and MK2, as well as attenuated RIPK1 S321 phosphorylation.

RIPK1 S321 phosphorylation also regulates RIPK1-induced apoptosis^21–23^. We determined whether TNF-induced apoptosis was affected by DAPK1 deficiency. Consistent with the inhibitory role of DAPK1 in RIPK1-mediated necroptosis, DAPK1 deficiency also conferred sensitivity to RIPK1-mediated apoptosis induced by TNF and AT-406 (Supplementary Fig. 10a). Suppression of either p38 MAPK or MK2 sensitized WT macrophages to RIPK1-mediated apoptosis (Supplementary Fig. 10b). Together, DAPK1-induced activation of p38 MAPK and MK2, leading to RIPK1 S321 phosphorylation, prevents TNF-triggered necroptosis and apoptosis.

### DAPK1 binds p38 MAPK and promotes p38 activation

MK2 is activated by p38 MAPK in the nucleus, followed by cytoplasmic entry to phosphorylate target substrates^44,45^. We determined the distribution of MK2 in *Dapk1*^*−/−*^ macrophages before and after TNF stimulation. The overall quantity of MK2 in the cytosol and nucleus was comparable between WT and *Dapk1*^*−/−*^ macrophages (Supplementary Fig. 11). The reduced phosphorylation of MK2 in *Dapk1*^*−/−*^ macrophages was thus not associated with altered cytoplasmic presence of MK2.

**Fig. 6:**
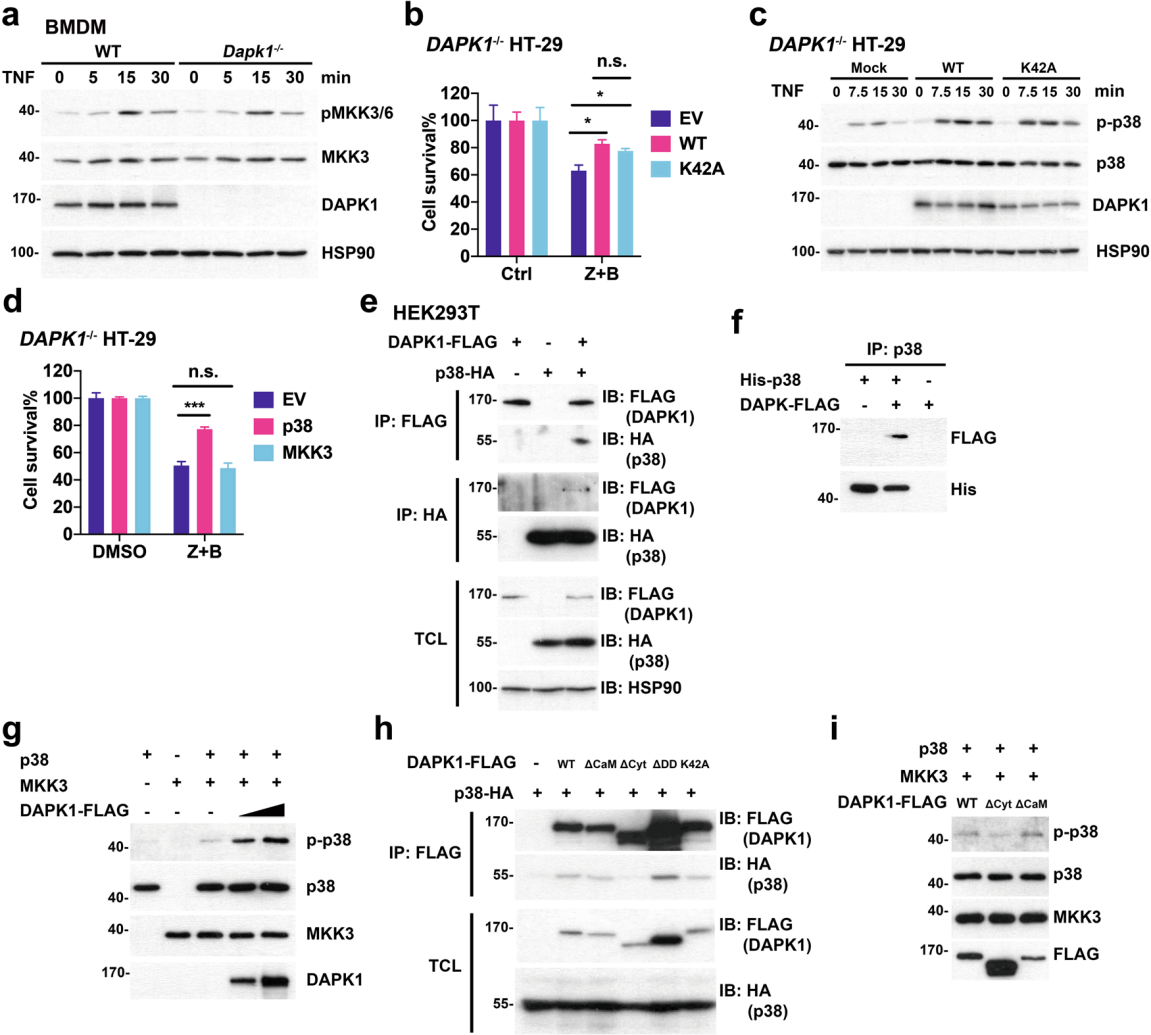
DAPK1-deficiency selectively inhibits phosphorylation of RIPK1(S321), MK2, and p38 MAPK. **a** DAPK1-deficiency does not affect MKK3 activation. WT and *Dapk1*^*−/−*^ BMDMs were treated with TNF, and levels of pMKK3 and MKK3 were determined in cell lysates. **b** DAPK1[K42A] increases resistance to necroptosis in DAPK1-deficient cells. *DAPK1*^−/−^ HT-29 cells were transfected with empty vector (EV), or transfected with WT DAPK1 or DAPK1[K42A]. Cells were treated with zVAD (Ctrl) or zVAD + BV6, and viability determined by ATP assay. **c** Either WT DAPK or DAPK1[K42A] increases p38 MAPK activation in DAPK1-null cells. *DAPK1*^−/−^ HT-29 cells, mock transduced, transduced with WT DAPK1 or DAPK1[K42A] were treated with TNF (20 ng/ml) for the indicated time points, and the levels of DAPK1, p38 MAPK and phospho-p38 MAPK were determined. **d** Overexpression of p38 MAPK inhibits necroptosis. *DAPK1*^−/−^ HT-29 cells were transfected with empty vector (EV), p38 MAPK or MKK3. The extent of necroptosis induced by zVAD+BV6 was determined by ATP release. **e** Interaction between DAPK1 and p38 MAPK. HEK293T cells were transfected with DAPK1-FLAG and/or p38-HA as indicated. Cell lysates were prepared 24 later, and precipitated with anti-FLAG or anti-HA. **f** p38 MAPK binds DAPK in vitro. Recombinant human p38 MAPK (200 ng) was incubated with purified DAPK (100 ng), as indicated. p38 MAPK was pulled down by anti-p38 MAPK (ab170099, Abcam), and the presence of DAPK and p38 MAPK in immunoprecipitants determined by anti-FLAG and anti-His, respectively. Results have been independently confirmed using another anti-p38 MAPK (7218, Cell Signaling). **g** DAPK1 promotes MKK3-directed p38 MAPK phosphorylation. Recombinant p38 MAPK (50 ng), MKK3 (100 ng), and DAPK1-FLAG (25 ng or 75 ng) was incubated as indicated in ATP-containing kinase buffer at 30 °C for 1 h. The amounts of p38 MAPK, MKK3, DAPK1 and phospho-p38 MAPK was determined by immunoblots. **h** Binding of different DAPK1 mutants to p38 MAPK. HEK293T cells were transfected with different mutants of DAPK1-FLAG and p38-HA as indicated. The presence of p38-HA in anti-FLAG precipitates was determined. **i** Failure of DAPK1(∆Cyt) to increase p38 MAPK activation. DAPK1, DAPK1(∆Cyt) or DAPK1(∆CAM) was evaluated for its ability to increase MKK3-directed p38 MAPK activation described in (**g**). Values are mean ± SD of triplicates in a single experiment. *P < 0.05, ***P < 0.001 (**b**, **d**) for two-way ANOVA followed by a Tukey’s multiple comparison test. Results have been repeated in three (**a**–**c**, **e**, **f**) or two (**d**, **g**–**i**) independent experiments.

We investigated how DAPK1 affects p38 MAPK activation. TNF-induced MKK3 phosphorylation was comparable between WT and *Dapk1*^*−*/−^ BMDMs (Fig. 6a), the attenuated TNF-triggered p38 MAPK activation in *Dapk1*^−/−^ BMDMs therefore suggest that p38 MAPK is the stage downstream of TNFR which is regulated by DAPK1.

We found that the kinase activity of DAPK1 was not involved in necroptosis inhibition. The introduction of kinase-dead DAPK1 mutant (DAPK1[K42A]) was as effective as WT DAPK1 in enhancing the survival of DAPK1-null HT-29 cells to necroptosis (Fig. 6b), as well as increasing the activation of p38 MAPK (Fig. 6c). Collectively, the kinase activity of DAPK1 is not required for conferring the resistance to necroptosis.

Introduction of p38 MAPK into DAPK1^*−*/−^ HT-29 cells increased the resistance to necroptosis induction (Fig. 6d). Overexpression of MKK3 alone, however, did not increase the survival of DAPK1-null HT-29 cells to necroptosis. In addition, DAPK1 was found to interact with p38 MAPK in HEK293T cells, in which p38 MAPK-HA was pulled down by DAPK1-FLAG, and vice versa (Fig. 6e). Recombinant p38 MAPK also bound purified DAPK1 protein (Fig. 6f), suggesting that DAPK1 may affect p38 MAPK activation through binding p38 MAPK. This was confirmed in an in vitro kinase analysis, in which recombinant MKK3 was used to activate recombinant p38 MAPK. The addition of increasing amounts of recombinant DAPK1 increased MKK3-directed p38 MAPK phosphorylation (Fig. 6g). In contrast, DAPK1[∆Cyt], the DAPK1 mutant lacking cytoskeleton-interacting domain, failed to bind p38 MAPK (Fig. 6h), and was less effective to activate p38 MAPK than WT DAPK1 (Fig. 6i). Therefore, DAPK1 selectively increases p38 MAPK activation likely by its specific interaction with p38 MAPK.

Together, our results illustrate that DAPK1 inhibits necroptosis induction through enhanced activation of p38-MK2-RIPK1 cascade. DAPK1 specifically targets the activation of p38 MAPK by binding p38 MAPK, with consequently increased activation of MK2 and elevated RIPK1 S321 phosphorylation, resulting in suppression of necroptosis.

## Discussion

In this study, we identified an unexpected role for DAPK1 in necroptosis. DAPK1 has mostly been shown to promote cell death. It mediates the apoptotic cell death induced by stimuli as diverse as Fas, TGF-β, ceramide, and ER stress^30–33,36^. This was also confirmed in the present study in which *Dapk1*^*−/−*^ BMDMs and DAPK1-knockdown Jurkat T lymphoma cells were more resistant than their WT counterparts to FasL- or ER stress-triggered death (Supplementary Fig. 5). Furthermore, DAPK1 mediates cell death induced by oxidative stress through phosphorylation of PKD and subsequent activation of JNK^43^, and during ischemic injury, by activation of the *N*-methyl-d-aspartate (NMDA) glutamate receptor through interaction and phosphorylation of the NR2B subunit^46^. DAPK1 has also been implicated in mediating autophagic cell death^36,47,48^. In contrast, DAPK1-deficiency rendered myeloid cells and HT-29 cells more sensitive to necroptosis triggered by treatment with zVAD plus either SMAC mimetics, TNF, or IFN-β (Figs. 1 and 2, Supplementary Figs. 2–4). The ability of DAPK1 to antagonize necroptosis therefore stands out among the capacity of DAPK1 to promote various types of cell death.

DAPK1 targets an early stage of the necroptotic pathway. At least three distinct kinases (IKKα/IKKβ, TBK1 and MK2) have been shown to directly phosphorylate RIPK1, thereby preventing formation of the RIPK1-containing death complex^20–25^. Our findings herein show that DAPK1 regulates inhibitory phosphorylation of RIPK1 at S321 (Fig. 5d). Moreover, DAPK1-deficiency led to diminished TNF-induced p38 MAPK activation, accompanied by reduced MK2 activation and decreased RIPK1 S321 phosphorylation (Fig. 5d). Inhibition of the activation of either p38 MAPK or MK2 conferred the susceptibility to necroptosis induction in WT macrophages (Fig. 5g). Attenuated p38/MK2 activation in *Dapk1*^*−/−*^ BMDMs also increased the sensitivity to necroptosis triggered by SMAC mimetic alone (Fig. 1f). The ability of DAPK1 to inhibit RIPK1 S166 phosphorylation was also demonstrated in the enhanced RIPK1 pS166-mediated apoptosis in *Dapk1*^*−/−*^ BMDMs (Supplementary Fig. 10a). Therefore, enhanced formation of the FADD–caspase 8–RIPK1–RIPK3 complex (Fig. 4) is partly attributable to reduced activation of p38 MAK/MK2 and attenuated phosphorylation of RIPK1 at S321 in DAPK1-deficient cells.

In addition to phosphorylation of the target proteins, DAPK1 has also been shown to regulate target protein function though protein-protein interaction that is independent of DAPK1 catalytic activity. DAPK1 binds and activates pyruvate kinase M2 (PKM2) in the absence of DAPK1 kinase domain^49^. The interaction of DAPK1 death domain with microtubule-affinity regulating kinase (MARK) activates MARK1/2 independent of DAPK1 kinase activity^50^. DAPK1 binds NLRP3 to promote NLRP3 inflammasome activation without the involvement of DAPK1 catalytic activity^51^. In the present study, we also found that the kinase activity of DAPK1 was not essential for repressing necroptosis (Fig. 6b, c). We further found that DAPK1-deficiency specifically attenuated the activation p38 MAPK, but not of ERK and JNK (Fig. 5c–e). In addition, the activation of MKK3 was normal in *Dapk1*^*−/−*^ BMDMs (Fig. 6a), suggesting the process regulated by DAPK1 is at the stage of p38 MAPK. Overexpression of p38 MAPK, but not MKK3, restored the viability of DAPK1^*−*/−^ HT-29 cells to necroptosis induction (Fig. 6d). A possible scaffold role of DAPK1 was suggested by the direct interaction of DAPK1 with p38 MAPK (Fig. 6e, f). We also mapped the cytoskeleton-binding domain of DAPK1 as the region interacting with p38 MAPK (Fig. 6h). Furthermore, in the in vitro kinase analysis containing only p38 MAPK, MKK3 and ATP, the inclusion of DAPK1 enhanced the phosphorylation of p38 MAPK (Fig. 6g). DAPK1 mutant (DAPK1∆Cyt) that did not bind p38 MAPK failed to enhance p38 MAPK activation in the same assay (Fig. 6h, i), suggesting that association of p38 MAPK with DAPK is required for the full TNFR-induced p38 MAPK activation. Therefore, our results provided the most direct evidences to illustrate on how DAPK1 increases TNF-directed p38 MAPK activation.

DAPK1 displays anti-inflammatory and pro-inflammatory activities in different cell types^52^. We previously demonstrated that TCR-induced NF-κB activation is enhanced in *Dapk1*^*−/−*^ T cells^42,53^, whereas LPS-triggered NF-κB activation is modestly reduced in *Dapk1*^*−/−*^ BMDMs^51^. In the present study, TNF-induced NF-κB activation and IKK activation in myeloid cells was not affected by DAPK1-deficiency (Fig. 5c), suggesting that increased necroptosis in *Dapk1*^*−/−*^ BMDMs is not linked to the processes of NF-κB activation. As another example, TCR-induced p38 MAPK activation is normal in *Dapk1*^*−/−*^ T cells^42^, while TNFR-initiated p38 MAPK phosphorylation was impaired in *Dapk1*^*−/−*^ BMDMs (Fig. 5d). These results further support the notion that signaling modulation by DAPK1 is cell type- and surface receptor-dependent^52^.

Necroptosis plays a dual role in cancer. Induction of necroptosis is a legitimate approach to killing tumor cells, especially for those that are resistant to apoptotic death, as confirmed by various in vitro studies^26,54,55^. Notably, RIPK1, RIPK3 and/or MLKL are downregulated in various types of cancer^56–58^, reflecting the necessity for tumor cells to circumvent necroptosis^26,59^. Our observation that DAPK1-deficiency increases necroptosis may seem incompatible with DAPK1’s tumor-suppressing role. However, our results also suggest that some of DAPK1’s inhibitory activity lies at the apex of the necroptotic pathway, i.e., RIPK1 S321 phosphorylation, but many tumors evade necroptosis at the effector stages, e.g. by mutations in RIPK3 or MLKL^26,54,55^. In addition, necroptosis may contribute to tumorigenesis^54,59^. RIPK1-RIPK3 necrosomes promote oncogenesis and immune suppression in pancreatic ductal adenocarcinoma^60^. Melanoma cells also trigger necroptosis of endothelial cells to promote tumor extravasation and metastasis^61^. Therefore, the necroptosis-inhibitory activity of DAPK1 does not necessarily contradict its tumor-suppressing function. The application of DAPK1-mediated necroptosis suppression is likely tumor-type and tumor stage dependent. Whether downregulation of DAPK1 in several tumor types may provide an opportunity to target these cancers with necroptosis-inducing agents warrants further exploration.

Necroptosis plays a critical role in the regulation of infection, inflammation and carcinogenesis, and the therapeutic applications of precise necroptosis regulation are well recognized. Recent studies reveal a specific role for RIPK1 phosphorylation at S321/326 in the control of necroptosis^21–23^. Our study suggests the possibility of regulating phosphor-S321/326 RIPK1-mediated necroptosis by modulating DAPK1 levels. Enhanced expression of DAPK1 inhibits necroptosis (Fig. S7), whereas reduced DAPK1 promotes necrosome formation (Figs. 1 and 2). Thus, DAPK1-mediated controlled necroptosis represents a potential therapeutic approach, but future research is needed to identify a reliable means of DAPK1-based necroptotic regulation.

The identification of p38 MAPK-MK2 as target signal molecules by DAPK1 reveals an additional unexpected regulatory pathway. MK2 has been implicated for its tumorigenic role in various cancers^62^. In addition, MK2 directly promotes autoimmune and inflammatory diseases including rheumatoid arthritis, chronic obstructive pulmonary disease, cardiovascular diseases and diabetes^63,64^. The selective association of DAPK1 to p38 MAPK-MK2 activation in macrophages provides a prospect in the regulation of MK2 activation and MK2-associated inflammatory pathology. Whether DAPK1 downregulation could be used to treat diseases mediated by p38-MK2 over-activation also deserves further investigation.

## Materials and methods

### DAPK1 expression and knockout

Human DAPK1-specific siRNA (siDAPK1; UCU GGG AAG CGG AGC UGA AUU) and siRNA control (siCtrl) were purchased from GE Dharmacon (Lafayette, CO, USA). *Dapk1*^−/−^ mice (in a C57BL/6 background) were previously described^36^. Mice were maintained in the SPF mouse facility of the Institute of Molecular Biology, Academia Sinica. All mouse experiments were conducted with approval from the Institutional Animal Care & Utilization Committee, Academia Sinica.

All mice used in this study were 8–12-week old. The same sex (male or female) mice were used in the same experiment, but opposite sex mice could be used in the repeat of the given experiment. No difference was observed between male and female mice in the analyses conducted in this study. Experimental groups were assigned randomly. Five or more mice in each experimental group was planned, but four mice in some experimental groups, that have been examined in previous studies, were used due to the knockout-mice availability. No blinding was done because the readouts of the mouse experiments in this study were clear-cut (body weight loss, death). No mice were excluded from scoring.

### Reagents

Purified LPS was purchased from InvivoGen (San Diego, CA). AT-406 was synthesized as described^65^. Flag-M2 antibodies, Flag-M2 beads, necrostatin-1 (Nec-1), cycloheximide (CHX), thapsigargin (THAP), propidium iodide (PI) and (3-(4,5-dimethylthiazol-2-yl)-2,5-diphenyl tetrazolium bromide) (MTT) were purchased from Sigma (St. Louis, MO). JNK inhibitor II (SP600125) and DMSO were purchased from Merck Millipore (Billerica, MA). Annexin V-Cy5 was obtained from BD-Biosciences (Franklin Lakes, NJ). Recombinant GM-CSF was purchased from R&D (Minneapolis, MN, USA). z-VAD-FMK was obtained from Bachem (Bubendorf, Switzerland). Recombinant mouse and human TNF were purchased from PeproTech (Rocky Hill, NJ, USA). Recombinant mouse IFNβ was purchased from PBL Assay Science (Piscataway, NJ, USA). BV6 was purchased from Selleck Chemicals (Houston, TX). Recombinant human Fas ligand (FLAG-FasL) was purchased from Enzo Life Sciences (Farmingdale, NY, USA). CellTiter-Glo^®^ Luminescent Cell Viability Assay was purchased from Promega (Fitchburg, Wisconsin). Lipofectamine 2000 was purchased from Thermo Fisher Scientific (Waltham, MA, USA). Constructs of p38α MAPK and active MKK3 (MKK3b(Glu^189^, Glu^193^)) were gifts of Dr. Jiahuai Han (Xiamen University, China).

### Antibodies

Anti-DAPK1 (D2178, DAPK-55, Western blot (WB) 1:1000), anti-HA (H3633, HA-7, WB 1:1000, immunoprecipitation (IP) 1 μg per test) and anti-FLAG (F1804, M2, WB 1:1000, IP 1 μg per test) were purchased from Sigma (St. Louis, MO). Anti-cIAP2 (sc-7944, H-85, WB 1:1000), anti-human FADD (sc-5559, H-181, IP 1ː500), anti-mouse FADD (sc-6036, M-19, IP 1:500), anti-human caspase-8 (sc-6136, C-20, IP 1:500), anti-AKT (sc-5298, B-1, WB 1:1000), anti-ERK (sc-154, C-14, WB 1:4000), anti-p38 MAPK (sc-535-G, C-20, WB 1:1000), anti-IκBα (sc-371, C-21, WB 1:2000) and anti-GAPDH (sc-32233, 6C5, WB 1:10000) were all purchased from Santa Cruz Biotech (Santa Cruz, CA). Anti-cIAP1 (GTX110087, WB 1:6000) was purchased from GeneTex (Irving, CA). Anti-human phospho RIPK1^S166^ (65746, D1L3S, WB 1:1000), anti-mouse phospho RIPK1^S166^ (31122, WB 1:1000), anti-human RIPK3 (13526, E1Z1D, WB 1:4000), anti-mouse RIPK3 (95702, D4G2A, WB 1:6000), anti-human caspase-8 (9746, 1C12, WB 1:500), anti-mouse caspase-8 (4927, WB 1:1000), anti-phospho AKT^S473^ (9271, WB 1:1000), anti-JNK (9252, WB 1:1000), anti-phospho JNK^T183/Y185^ (9251, WB 1:1000), anti-phospho ERK^T202/Y204^ (9101, WB 1:4000), anti-phospho IκBα^S32/S36^ (9246, 5A5, WB 1:1500), anti-phospho IKKα^S176^/IKKβ^S177^ (2078, C84E11, WB 1:1000), anti-phospho TBK1^S172^ (5483, D52C2, WB 1:1000), anti-phospho RIPK1^S321^ (83613, WB 1:2000), anti-phospho p38^T180/Y182^ (9211, WB 1:1000), anti-phospho MK2^T334^ (3007, WB 1:500), anti-IKKβ (8943, D30C6, WB 1:1000), anti-TBK1 (3504, WB 1:1000), anti-MK2 (3042, WB 1:1000) and anti-Myc (2276, 9B11, WB 1:1000, IP 1 μg per test) were all purchased from Cell Signaling Technology (Danvers, MA). Anti-mouse RIPK3 (2283, WB 1:6000) was purchased from ProSci (San Diego, CA). Anti-human phospho RIPK3^S227^ (ab209384, EPR9627, WB 1:4000), anti-mouse phospho RIPK3^S232^ (ab195117, EPR9516(N)-25, WB 1:3000), anti-phospho p38^T180/Y182^ (ab170099), anti-human MLKL (ab184718, EPR17514, WB 1:6000), anti-human phospho MLKL^S358^ (ab187091, EPR9514, WB 1:6000), anti-mouse phospho MLKL^S345^ (ab196436, EPR9515(2), WB 1:6000) and anti-mouse caspase-8 (ab138485, IP 1:500) antibodies were purchased from Abcam (Cambridge, UK). Anti-FADD (05-486, 1F7, WB 1:1000) and anti-mouse MLKL (MABC604, 3H1, WB 1:2000) were purchased from Merck Millipore (Billerica, MA). Anti-RIPK1 (610459, 38/RIP, WB 1:6000), anti-HSP90 (610419, 68/Hsp90, WB 1:4000), anti-CD3-PE (145-2C11, flow cytometry (FCM) 1:100), anti-Gr-1-PE (RB6-8C5, FCM 1:100) and anti-I-Ab-PE (AF6-120.1, FCM 1:100) were obtained from BD-Biosciences (Franklin Lakes, NJ). Anti-CD4-PE (RM4-4, FCM 1:100), anti-B220-FITC (RA3-6B2, FCM 1:100), anti-CD11b-PE-Cy7 (M1/70, FCM 1:100), anti-CD11c-PB (N418, FCM 1:100), anti-F4/80-APC (BM8, FCM 1:100) and anti-Fas-APC (DX2, 1:100) antibodies were purchased from BioLegend (San Diego, CA, USA). Anti-CD8-PE (53-6.7, FCM 1:100) and anti-Ly6C-APC (HK1.4, FCM 1:100) were purchased from eBioscience (San Diego, CA, USA).

### Cell culture

HEK293T (ATCC CRL-3216), HT-29 (ATCC HTB-38), J774A.1 (ATCC TIB-67) and Jurkat T lymphoma (clone E6-1, ATCC TIB-152) and L929 (ATCC CCL-1) cell lines were obtained from ATCC. Cell lines were examined for mycoplasma contamination using a Mycoplasma Detection Kit (R&D). HT-29 cells and Jurkat cells were cultured in complete RPMI-1640 medium containing 10% fetal calf serum (Invitrogen Life Technology), 10 mM glutamine, 100 U/ml penicillin, 100 μg/ml streptomycin and 50 μM 2-mercaptoethanol. HEK293T, J774A.1 and L929 cells were cultured in complete DMEM medium with the same supplements as for complete RPMI medium. Bone marrow cells were collected from tibias and femurs by flushing with cold phosphate buffered saline (PBS). Bone marrow cells were cultured in complete DMEM medium with 20% L929-conditioned media for 6 days to generate bone marrow-derived macrophages (BMDMs). Bone marrow cells were also differentiated in complete RPMI medium containing 20 ng/ml GM-CSF for 8 days to generate bone marrow-derived dendritic cells (BMDCs). DAPK was also knocked down in HT-29 cells by transfection with siDAPK1 using Lipofectamine 2000.

### Cell viability assay

Necroptosis was induced by pretreating cells with z-VAD for 0.5 h, followed by stimulation with AT406, BV6, LPS, TNF or IFNβ for the indicated periods. The necroptosis inhibitor Nec-1 was added 0.5 h prior to stimulation for certain experiments. Cell viability of BMDMs or BMDCs was assessed by measuring ATP levels upon adding an equal volume of Cell Titer-Glo reagent (Promega) and incubating for 30 min. Luminescence was determined using a Victor3 1420 Multilabel Counter (PerkinElmer, Shelton, CT). Alternatively, cell viability was determined via reduction of MTT by mitochondrial reductase into purple formazan. The intensity of colored product was measured by absorbance at 490 nm on an Emax microtiter plate reader (Molecular Device, Sunnyvale, CA). Necroptotic HT-29 cells were determined by staining with PI (10 μg/ml) in PBS, and PI^+^ cells were analyzed using flow cytometry. Apoptosis of Jurkat cells was induced by treating with FLAG-FasL and assessed by staining with Annexin V-Cy5 (BD-Biosciences) and subsequent Annexin V^+^ cell quantitation by flow cytometry.

### Generation of *DAPK1* knockout HT-29 cell by CRISPR-Cas9 editing

*DAPK1*^−/−^ HT-29 cells were generated using an All-in-One Cas9^D10A^ nickase system^66^. AIO-GFP plasmid was purchased from Addgene (#74119). The specific DAPK1-sgRNA was designed using CRISPR Design (http://crispr.mit.edu/) at exon 2 of the *DAPK1* locus on chromosome 9. A pair of sense DAPK1-sgRNA was annealed using 5′ -accgTGA TTA CTA CGA CAC CGG CG-3′ and 5′-aaacCG CCG GTG TCG TAG TAA TCA-3′, and a pair of antisense DAPK-sgRNA was annealed using 5′-accgCAC GTT TTC CTG CCT GAA CA-3′ and 5′-aaacTG TTC AGG CAG GAA AAC GTG-3′ (Supplementary Fig. 5a). AIO-GFP plasmid containing DAPK1-sgRNA was transfected into HT-29 cells by electroporation using the MP-100 system (Life Technologies), and GFP^+^ cells were isolated by cell sorting. Sorted cells were sub-cultured into 96-well plates for single clone selection. Exon 2 of the *DAPK1* locus was amplified from individual clones by PCR and sequenced to verify its deletion.

### Western blot and immunoprecipitation

For immunoblotting and immunoprecipitation, cells were lysed by whole-cell extract (WCE) buffer (25 mM HEPES pH 7.9, 300 mM NaCl, 1.5 mM MgCl_2_, 0.2 mM EDTA, 0.5 mM DTT and 0.1% Triton X-100) on ice for 30 min. Supernatants were separated by centrifugation at 13,200 rpm for 10 min at 4 °C. The protein concentrations were determined by Bio-Rad protein assay (#500-00006). For immunoprecipitation, 0.5 mg total cell lysates were incubated with 1 μg specific antibody, and the mixtures were rotated overnight at 4 °C. The immune-complexes were captured by Protein G Mag Sepharose (GE, 28-9670-70, 10 μl per sample). The beads were washed three times with WCE buffer and denatured by 4X SDS sample buffer (200 mM Tris-HCl pH 6.8, 1.2 M β-mercaptoethanol, 40% glycerol, 8% SDS, and 0.4% bromophenol blue). For immunoblots, samples were denatured and analyzed by SDS-PAGE with running buffer (0.025 M Tris, 0.192 M glycine, and 0.1% SDS). The PAGE was transferred to PVDF membrane (Millipore) with transfer buffer (25 mM Tris, 192 mM glycine, and 20% methanol) at 400 mA for 100 min at 4 °C. Membranes were blocked with SuperBlock™ T20 (Sigma) to detect anti-phosphate antibody or blocking buffer (5% non-fat milk and 0.1% Tween-20 in a TBST buffer of 50 mM Tris-HCl pH 7.4 and 150 mM NaCl) at room temperature for 30 min, before being incubated with specific primary antibodies at indicated dilutions at 4 °C overnight. The membranes were washed three times with wash buffer (0.1% Tween-20 in TBST buffer) at room temperature for 10 min before incubating them with horseradish peroxidase-conjugated secondary antibodies in blocking buffer at room temperature for 1 h. After washing, the membranes were developed with ECL Western blot detection reagents (Advansta, K-12045-D50), with signals detected by X-ray film (Fujifilm).

### TNF-induced septic shock

Wild-type littermates or *Dapk1*-knockout C57BL/6 mice of 6–8 weeks and same sex were used for TNF-induced septic shock. No randomization was used due to the availability of knockout mice. Mice were anesthetized by Avertin (0.25 ml of 20 mg/ml, Sigma) and mouse TNF (1.0 μg/g) was intravenously administered in total volume of 200 μl endotoxin-free PBS. Body temperatures were monitored from rectal by industrial electric thermometer (Kane-may) for 30 h, with simultaneous recording of mice death. Mice were sacrificed when body temperature fell below 22 °C. No blinding was done because the readouts of septic shock were straightforward (temperature drop, death). No mice were excluded from scoring.

### In vitro kinase analysis

Recombinant human His-p38 MAPK (ab82188) and recombinant human His-MKK3 (ab105578) were obtained from Abcam. DAPK-FLAG protein was purified from cell lysates of HEK293T cells transfected with DAPK-FLAG, by anti-FLAG M2 affinity gel and eluted with 3× FLAG peptides. Recombinant p38 MAPK (50 ng) and recombinant MKK3 (100 ng) were incubated with purified DAPK-FLAG (0, 25 ng or 75 ng) in kinase buffer (200 μM ATP, 20 mM HEPES (pH 7.6), 20 mM MgCl_2_, 1 mM DTT) at 30°C for 1 h. The extent of p38 MAPK phosphorylation was determined.

### Statistics

Data were randomly collected, but not blindly. We did not exclude any data from this study. Data met the assumptions of applied statistical tests (i.e. normal distributions). Microsoft Office Excel and Prism 5.0 (GraphPad software) were used for data analysis. Unpaired two-tailed Student's *t* tests were used to compare most of the viability or death results between two groups. Two-way ANOVA followed by a Tukey’s multiple comparison tests, or two-way ANOVA followed by a Sidak’s multiple comparison test were used to compare other results, as indicated in the respective figure legends. Long-rank (Mantel–Cox) test was used to compare the survival of mice. Data are presented as means with standard deviation (SD).

## Supplementary information

Supplementary Figure Legends

Supplementary Figure 1

Supplementary Figure 2

Supplementary Figure 3

Supplementary Figure 4

Supplementary Figure 5

Supplementary Figure 6

Supplementary Figure 7

Supplementary Figure 8

Supplementary Figure 9

Supplementary Figure 10

Supplementary Figure 11
