# Supplementary material for: Tumor suppressor death-associated protein kinase 1 inhibits necroptosis by p38 MAPK activation
Source: Cell Death Dis. 2020 May 4;11(5):305. doi: 10.1038/s41419-020-2534-9 (PMC7198492; doi:10.1038/s41419-020-2534-9)
Supplement: Supplementary file 1 — Supplementary Figure Legends [file 41419_2020_2534_MOESM1_ESM.docx]

**Supplementary Figure 1. Normal myeloid cell populations in *Dapk1*^-/-^ mice.** (a) Splenocytes from WT and *Dapk1*^-/-^ mice were stained for indicated surface markers and determined by flow cytometry. (b) Bone marrow cells from WT and *Dapk1*^-/-^ mice were stained for indicated surface markers and determined by flow cytometry. Lin^-^ cells represent the CD3^-^CD4^-^CD8^-^CD11b^‑^B220^-^ cell population. Numbers in plots indicate percentages of each boxed/circled cell fraction. All data are representative of three independent experiments.

**Supplementary Figure 2. DAPK1-deficiency increases necroptosis, as measured by MTT uptake.** WT and *Dapk1*^-/-^ BMDMs were treated with combinations of 20 μM z-VAD and 0.6 μM AT-406 as indicated for 17-20 hours. Cell viability was assessed by incorporation of MTT. Values are mean ± SD of triplicates in a single experiment. ****P* < 0.001 for unpaired *t*-test. Results have been repeated in two independent experiments.

**Supplementary Figure 3. Loss of DAPK1 increases necroptosis in dendritic cells.** Bone marrow-derived dendritic cells from WT and *Dapk1*^-/-^ mice were treated with combinations of z-VAD (20 μM), AT-406 (0.5 μM), and Nec-1 (40 μM) for 18 h. Cell viability was assessed by measuring ATP levels. Values are mean ± SD of triplicates in a single experiment. ****P* < 0.001 for unpaired *t*-test. Results have been repeated in two independent experiments.

**Supplementary Figure 4. Enhanced necroptosis is DAPK1-specific.**  (a) Knockdown of DAPK1 in a J774A.1 macrophage cell line. J774A.1 cells were transduced with pLL3.7-shCtrl or pLL3.7-shDAPK1, sorted, and their expression of DAPK1 was then determined. (b) Increased zVAD-induced necroptosis in DAPK1-deficient J774A.1 cells. Control and DAPK1-knockdown J774A.1 cells were treated with DMSO, zVAD (20 μM), or zVAD + Nec-1 (40 μM, N) for 24 h before determining cell survival by MTT assay. (c) Re-introduction of DAPK1 to DAPK1-knockdown J774A.1 cells inhibits necroptosis. Control, DAPK1-deficient J774A.1, or human DAPK1-re-expressing J774A.1 cells were treated with DMSO, zVAD (5 μM), or zVAD + AT-406 (1 μM), and then cell survival was determined by MTT assay. Values are mean ± SD of triplicates in a single experiment. ****P* < 0.001 for unpaired *t*-test. Results have been repeated in three independent experiments.

**Supplementary Figure 5. DAPK1 participates in ER stress- and death receptor-induced apoptosis.** (a) WT and *Dapk1*^-/-^ BMDMs were treated with DMSO or 4 μM thapsigargin (THAP) for 17 hours. Cell viability was assessed by MTT assay. (b, c) Jurkat cells were transduced with pLL3.7-GFP-shCtrl or pLL3.7-GFP-shDAPK1, and GFP^+^ cells were isolated by cell sorting. Expression of Fas (CD95) was determined by flow cytometry (b). GFP control and DAPK1-knockdown Jurkat cells were treated with FasL for 4 h and apoptotic cells were quantified by Annexin-V staining (c). (d) WT and *Dapk1*^-/-^ BMDMs were treated with FasL, and cell survival was quantitated after 24 h. Values (a, c, d) are mean ± SD of triplicates in a single experiment. **P* < 0.05, ***P* < 0.01 for unpaired *t*-test. Results have been repeated in three independent experiments.

**Supplementary Figure 6. DAPK1 knockout enhances necroptosis in HT-29 cells.** (a) Sequences of the sense (S) and antisense (AS) sgRNAs were designed at exon 2 of the *DAPK1* locus on chromosome 9. Cas9^D10A^ nicking sites are indicated by red arrows (top panel). Genomic DNA was extracted from single HT-29 clones. Exon 2 of the *DAPK1* locus was then amplified by PCR, and the sequences were determined. Two DAPK1 knockout HT-29 clones were selected and the DNA sequences of the two alleles of DAPK1 in these clones are shown. Deletions are represented by dashed lines, and inserted sequences are tagged with black arrowheads (bottom clone). (b) The expressions of DAPK1, RIP1, RIP3, MLKL, and FADD were examined by Western blot from WT and DAPK1 knockout HT-29 cells. (c) WT and DAPK1 knockout HT-29 cells were treated with combinations of z-VAD (Z, 20 μM), BV6 (B, 0.5 μM), and Nec-1 (N, 40 μM) for 24 hours. Cell death was assessed by PI staining, with y-axis scale representing percentage of PI^+^ cells. Values are mean ± SD of triplicates in a single experiment. ****P* < 0.001 for unpaired *t*-test. (d) WT and DAPK1-knockout HT-29 cells were treated with z-VAD (20 μM) + BV6 (0.5 μM) for the indicated time-points and phosphorylation of RIPK1(S166), RIPK3, and MLKL were determined by Western blot. All data are representative of three independent experiments.

**Supplementary Figure 7. DAPK1 overexpression suppresses necroptosis in HT-29 cells.**

(a, b) HT-29 cells were transduced with TRIP-GFP or TRIP-GFP-DAPK1, and GFP^+^ cells were isolated by cell sorting. Expression of DAPK1 was determined by Western blot (a). GFP control and DAPK1-FLAG-expressing HT-29 cells were treated with z-VAD (20 μM) and TNF (100 ng/ml) for 48 hours. Cell death was assessed by PI staining (b). (c, d) *DAPK1*^-/-^ HT-29 cells were transduced with TRIP-GFP or TRIP-GFP-DAPK, and GFP^+^ cells were isolated by cell sorting. Expression of DAPK1 was determined by Western blot (c). GFP control and DAPK1-FLAG-expressing *DAPK1*^-/-^ HT-29 cells were treated with z-VAD (20 μM) plus BV6 (0.5 μM) for 48 hours. Cell death was assessed by PI staining (d). Values are mean ± SD of triplicates in a single experiment. ***P* < 0.01, ****P* < 0.001 unpaired *t*-test. Results have been repeated in two independent experiments.

**Supplementary Figure 8. DAPK1 deficiency does not affect SMAC mimetic-induced cIAP degradation**. (a) WT and *Dapk1*^-/-^ BMDMs were treated with zVAD (Z, 20 μM) plus AT-406 (A, 0.6 μM), and the contents of cIAP1 at the indicated time points determined by immunoblots. (b) Control and DAPK1-knockdown HT-29 cells were treated with BV6, and cIAP1 levels assessed at the indicated time points. Data are representative of two independent experiments.

**Supplementary Figure 9. Attenuated p38 MAPK activation and normal ERK phosphorylation in *Dapk1*^-/-^ BMDMs**. WT and *Dapk1*^-/-^ BMDMs were untreated or treated with TNF (20 ng/ml) for 15 min on cover slips, stained with anti-phospho-p38 (a), anti-phospho-ERK (b) and DAPI for confocal image acquisition. Scale bar, 20 μm. Quantification of Corrected total cell fluorescence (CTCF) from each confocal images were measured by using ImageJ software (right panel). RIPK1 was used to define the image area of each cell. Data are mean ± SD. n = 232 for WT and 251 for *Dapk1*^-/-^ (a), 109 for WT and 174 for *Dapk1*^-/-^ (b). ****P* < 0.001 from two-tailed Student’s *t*-test. Similar results were obtained from three independent experiments for (a) and (b).

**Supplementary Figure 10. Increased sensitivity of *Dapk1*^-/-^ BMDMs to RIPK1-mediated apoptosis**. (a) DAPK deficiency increased RIPK1-mediated apoptosis. WT and *Dapk1*^-/-^ BMDMs were treated with TNF+AT-406 and cell viability determined. (b) Suppression of p38 MAPK and MK2 activation confers susceptibility to RIPK1-mediated apoptosis in WT macrophages. WT BMDMs were treated with TNF, AT-406, SB203589 and PF3644022 as indicated, and viability quantitated. Values are mean ± SD of triplicates in a single experiment. ****P* < 0.001 for unpaired Student’s *t*-test. Results are representative of two independent experiments.

**Supplementary Figure 11. DAPK1 deficiency does not affect the distribution of MK2**. WT and *Dapk1^-/-^* BMDMs were treated with TNF (20 ng/ml), and nuclear extracts and cytosolic extracts were isolated at the indicated time-points to determine the contents of phospho-MK2 and MK2. GAPDH and HDAC were used as marker for cytoplasm and nucleus, respectively. Data are representatives of two independent experiments.
